# Performance of a blockwise approach in variable selection using linkage disequilibrium information

**DOI:** 10.1186/s12859-015-0556-6

**Published:** 2015-05-08

**Authors:** Alia Dehman, Christophe Ambroise, Pierre Neuvial

**Affiliations:** Laboratoire de Mathématiques et Modélisation d’Evry (LaMME), Université d’Evry-Val-d’Essonne/UMR CNRS 8071/ENSIIE/USC INRA, Evry, France

**Keywords:** Genome-wide association studies, Linkage disequilibrium, Hierarchical clustering, Model selection, Gap statistic, Penalized regression, Group lasso

## Abstract

**Background:**

Genome-wide association studies (GWAS) aim at finding genetic markers that are significantly associated with a phenotype of interest. Single nucleotide polymorphism (SNP) data from the entire genome are collected for many thousands of SNP markers, leading to high-dimensional regression problems where the number of predictors greatly exceeds the number of observations. Moreover, these predictors are statistically dependent, in particular due to linkage disequilibrium (LD).

We propose a three-step approach that explicitly takes advantage of the grouping structure induced by LD in order to identify common variants which may have been missed by single marker analyses (SMA). In the first step, we perform a hierarchical clustering of SNPs with an adjacency constraint using LD as a similarity measure. In the second step, we apply a model selection approach to the obtained hierarchy in order to define LD blocks. Finally, we perform Group Lasso regression on the inferred LD blocks. We investigate the efficiency of this approach compared to state-of-the art regression methods: haplotype association tests, SMA, and Lasso and Elastic-Net regressions.

**Results:**

Our results on simulated data show that the proposed method performs better than state-of-the-art approaches as soon as the number of causal SNPs within an LD block exceeds 2. Our results on semi-simulated data and a previously published HIV data set illustrate the relevance of the proposed method and its robustness to a real LD structure. The method is implemented in the R package BALD (Blockwise Approach using Linkage Disequilibrium), available from http://www.math-evry.cnrs.fr/publications/logiciels.

**Conclusions:**

Our results show that the proposed method is efficient not only at the level of LD blocks by inferring well the underlying block structure but also at the level of individual SNPs. Thus, this study demonstrates the importance of tailored integration of biological knowledge in high-dimensional genomic studies such as GWAS.

**Electronic supplementary material:**

The online version of this article (doi:10.1186/s12859-015-0556-6) contains supplementary material, which is available to authorized users.

## Background

With recent advances in high-throughput genotyping technology, genome-wide association studies (GWAS) have become a tool of choice for identifying genetic markers underlying a variation in a given phenotype – typically complex human diseases and traits. In GWAS, information on genetic polymorphisms is collected across the genome and single nucleotide polymorphisms (SNPs) are typically used due to their abundance in the genome. However, common genetic variants identified by GWAS only account for a relatively small proportion of the heritability of diseases [1].

The most widely used approach for selecting causal SNPs is to perform univariate tests of association between the phenotype of interest and the genotype of each marker [2,3]. Following [4], this type of approach will be referred to as Single Marker Analysis (SMA). The results of SMA are often refined in two-ways. First, due to linkage disequilibrium (LD) between SNPs, combining the *p*-values obtained by SMA into gene-level statistics may yield more interpretable results [5]. Second, candidate markers selected by SMA may be incorporated into a *multi-variable linear models of association*. Recent studies suggest that penalized regression methods such as Lasso [6] and Elastic-Net [7] may be appropriate to identify the additive effect of several genetic markers [4,8-10]. Such methods allow multi-variable linear models to be estimated in high-dimensional situations such as GWAS, where the number *p* of variables (i.e., SNP markers) exceeds the number *n* of observations (i.e., individuals) by several orders of magnitude. In this paper, we propose a penalized regression approach tailored to the dependence between markers in GWAS induced by linkage disequilibrium (LD). Our goal is to identify common variants which may have been missed by SMA because their individual effect size is not large enough to pass genome-wide significance thresholds.

**Figure 1:**
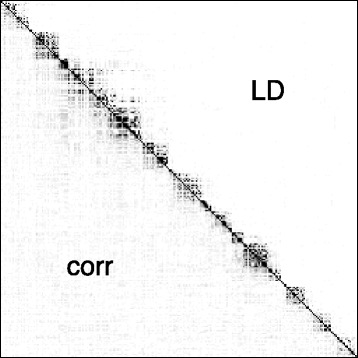
Blockwise dependency in real genotyping data: 256 SNPs spanning the first 1.45 Mb of Chromosome 6 in [11]. The average distance between two successive SNPs is approximately 5 kb. The upper triangular part of the matrix displays measures of LD (*r* ^2^ coefficients) between pairs of SNPs, while its lower triangular part displays absolute sample correlations between pairs of SNP genotypes. Colors range linearly from 0 (white) to 0.4 (black).

As a motivating example for our contribution, Figure 1 represents the LD (*r* ^2^ coefficients, upper triangular part) and the sample genotype correlations (lower triangular part) between the first 256 SNPs of chromosome 6 in a study on 605 HIV-infected patients [11]. A blockwise structure can be distinguished, where the average LD within blocks of 12 to 15 SNPs is approximately *r* ^2^=0.2. The LD values are notably more contrasted than the correlation values, as many *r* ^2^ coefficients are very close to 0. In order to account for, and take advantage of this strong dependency structure between adjacent or nearby SNPs, it makes sense to focus on the scale of LD blocks, and to explicitly look for *sets of LD blocks jointly associated to the phenotype of interest*.

In order to do so, we propose a three-step method which consists in (i) inferring groups of SNP –that is, LD blocks– using a spatially-constrained hierarchical clustering algorithm, (ii) applying a model selection approach to estimate the number of groups, and (iii) identifying associated groups of SNPs using a Group Lasso regression model [12]. This approach is described in Section ‘A three-step method for GWAS’. Sections ‘Implementation’, ‘Competing methods’, ‘Performance evaluation’ and ‘SNP and block-level evaluation’ cover a description of its implementation and of the evaluation methods used for performance assessment. In Sections ‘Results on simulated data’ and ‘Results on semi-simulated data’, the proposed method is compared to state-of-the-art competitors on simulated and semi-simulated data. Section ‘Analysis of HIV data’ describes the application of the proposed method on microarray data from a specific GWA study on HIV.

## Methods

### A three-step method for GWAS

The problem of selecting causal SNPs can be cast as a problem of high-dimensional variable selection. We consider the problem of predicting a continuous response $\mathbf {y} \in \mathbb {R}^{n}$ from covariates $\mathbf {X} \in \mathbb {R}^{n \times p}$. For *i*∈{1,…,*n*}, **X**_*i*·_ is a *p*-dimensional vector of covariates for observation *i* and for *j*∈{1,…,*p*}, **X**_·*j*_ is a *n*-dimensional vector of observations for covariate *j*. In GWAS, the covariates are ordinal and correspond to SNP genotypes: *X*_*ij*_∈{0,1,2} correspond to the number of minor alleles at locus *j* for observation *i*. For each *i*∈{1,…,*n*}, we assume that **X**_*i*·_ has a block structure with *G* non-overlapping blocks of sizes *p*_1_,…*p*_*G*_, with $\sum _{g=1}^{G}{p_{g}}=p$. Thus $\mathbf {X}_{i \cdot }=(\mathbf {X}_{i \cdot }^{1}, \dots, \mathbf {X}_{i \cdot }^{G})$ with $\mathbf {X}_{i \cdot }^{g} \in \mathbb {R}^{p_{g}}$ for *g*=1,…,*G*.

We propose a three-step method consisting in (i) performing a spatially constrained hierarchical clustering of the covariates **X**, (ii) estimating the number of groups using (a modified version of) the Gap statistic [13], and (iii) performing a Group Lasso regression to identify which of the inferred groups are associated with the response **y**.

#### Inference of LD blocks from genotypes

The first step of the proposed approach consists in inferring LD blocks using a spatially constrained hierarchical clustering algorithm. Only the genotype data **X** are used at this step.

The proposed clustering procedure is based on the one of the most widely used methods for cluster analysis: Ward’s incremental sum of squares algorithm [14]. The general goal of sum of squares clustering is to minimize the total within-group dispersion *W*_*G*_ for a given number of groups *G*. Denoting by $S_{gg'}\phantom {\dot {i}\!}$ the total squared similarity (usually, the similarity induced by the Euclidean distance) between all pairs of items in groups *g* and *g* ^′^, and by *p*_*g*_ the size of group *g*, *W*_*G*_ may be written as

$$\begin{array}{@{}rcl@{}} W_{G} = p - \sum_{g=1}^{G}{\frac{S_{gg}}{p_{g}}}\,. \end{array} $$

The standard agglomerative hierarchical clustering starts with *p* groups of size 1 and successively merges the pair of groups *g* and *g* ^′^ leading to the minimal increase in within-group dispersion. Equivalently, this corresponds to merging the two closest groups *g* and *g* ^′^ that minimize the following distance:

$$\begin{array}{@{}rcl@{}} d_{g,g'} & = & \frac{p_{g} p_{g'}}{p_{g} + p_{g'}} \left(\frac{S_{gg}}{{p_{g}^{2}}} +\frac{S_{g'g'}}{p_{g'}^{2}} - \frac{2S_{gg'}}{p_{g} p_{g'}} \right) \,. \end{array} $$

This merging process is repeated until a single group of size *p* remains.

Our proposed clustering algorithm differs in two respects. First, instead of the standard Euclidean distance, we use a measure of the dissimilarity between two SNPs *j* and *j* ^′^ based on LD: 1 − *r* ^2^(*j*,*j* ^′^). This is made possible by the fact that the above-described algorithm depends on the genotype data **X** only through the matrix of pairwise similarities between variables (by the definition of $\phantom {\dot {i}\!}S_{gg'}$); this can be viewed as an instance of *kernel trick* [15]. Second, we take advantage of the fact that the LD matrix can be modeled as block-diagonal (see Figure 1) by only allowing groups of variables that are *adjacent on the genome* to be merged.

#### Estimation of the number of groups

The choice of the number of groups *G* is often ambiguous and depends on many parameters of the data set. Any choice of *G* corresponds to a tradeoff between compressing the data into few groups or reducing the amount of error by increasing the number of groups. Several model selection criteria have already been investigated to make such a decision [13,16-18]. All of these methods are based on the above-defined measure of within-group dispersion (*W*_*G*_)_*G*=1…*p*_.

We have chosen to use a modified version of the Gap statistic [13] as a model selection criterion. The Gap statistic compares *W*_*G*_ to its expectation under an appropriate reference null distribution of the data. For a clustering into *G* groups, we calculate the following quantity:

(1) $$\begin{array}{@{}rcl@{}}  \text{Gap}^{\star}(G)= \frac{1}{B}\sum_{b=1}^{B} \left({W^{b}_{G}} - W_{G}\right), \end{array} $$

where for *b*=1…*B*, ${W^{b}_{G}}$ denotes the within-cluster dispersion of clustering the reference data set *b* in *G* groups. For each reference data set, each column is drawn independently from a uniform distribution over the range of observed values for this column. In applications to GWAS, this corresponds to a uniform distribution over the discrete set of observed genotypes {0,1,2}. Thus, the reference data sets correspond to data with no structure among the variables. We define the optimal number of groups $\hat {G}$ as the smallest *G* such that Gap^⋆^(*G*)≥Gap^⋆^(*G* + 1)−*s*_*G*+1_, where *s*_*G*_ is a standard error estimate calculated as *s*_*G*_=(1+*B*)^−1/2^sd_*G*_, where sd_*G*_ denotes the standard deviation of $({W_{G}^{b}})_{1 \leq b \leq B}$. In the classical version of the Gap statistic, the logarithm of *W*_*G*_ is used instead of *W*_*G*_, and several alternatives to this original definition have been investigated recently [19]. We decided to use the definition in Equation 1 as we noticed that it led to better estimation of the number of groups in our simulation studies, which were performed under a variety of parameters and on several data sets. For the reference distribution, we followed the initial strategy proposed in the original Gap statistic paper [13] and simulated each reference feature according to a uniform distribution over the discrete set {0,1,2}. We chose to simulate *B*=100 reference samples since we empirically observed that it was sufficient to provide a stable estimation of the number of groups.

#### Selection of groups associated with the response

Once LD blocks have been identified, we use Group Lasso regression [12] to identify blocks associated with the phenotype. Well adapted to group-structured variables, the Group Lasso estimator is defined as:

$$ \begin{array}{rcl}{\widehat{\boldsymbol{\beta}}}_{\mathrm{GL}}& =& {arg\; min}_{\beta \in {\mathbb{R}}^p}\left(\left|\right|\mathbf{y}-\mathbf{X}\boldsymbol{\beta } \left|\right|{}_2^2+\lambda \sum_{g=1}^G\sqrt{p_g}\left|\right|{\boldsymbol{\beta}}^g\left|\right|{}_2\right),\end{array} $$

where ||.||_2_ denotes the Euclidean norm, *λ* is a penalty parameter, and ***β*** ^*g*^ denotes the *p*_*g*_-dimensional vector of regression coefficients corresponding to the *g* ^th^ group, so that ***β***=(***β*** ^1^,…***β*** ^*G*^). The Group Lasso is a group selection method: by construction, the estimated coefficients within a group tend to be either all zero or all nonzero. In practice, the columns of the design matrix *X* are scaled before performing Group Lasso regression.

### Implementation

The proposed three-step method has been implemented in an R package called BALD for “Blockwise Approach using Linkage Disequilibrium”. This package is available from http://www.math-evry.cnrs.fr/publications/logiciels. We have used the packages grplasso for Group Lasso regression and quadrupen for Lasso and Elastic-Net regression [20], both of which are available from CRAN at http://cran.r-project.org.

A naive implementation of the constrained clustering described in Section ‘Inference of LD blocks from genotypes’ would consist in (i) calculating the *p*(*p*−1)/2 LD measures for each pair of SNP and (ii) performing constrained hierarchical clustering on the obtained similarity matrix. As *p* is typically of the order of 10^4^ to 10^5^ for a single chromosome in a standard GWAS, such an implementation with space complexity of *O*(*p* ^2^) is not appropriate. Indeed, for a single chromosome of length *p*=10^5^, this algorithm would require storing of the order of 10^10^ numeric values of LD before applying the clustering algorithm. To overcome this difficulty, our implementation takes as input the *n*×*p* matrix of genotypes **X**, and calculates the LD measures incrementally as they are required by the clustering. The LD measures are calculated directly from genotypes using the Bioconductor R package snpStats [21,22], which handles missing values. For illustration, it takes 4.5 hours (on a standard 2.2 Ghzsingle CPU) to analyze a whole genome of 500*k* simulated SNPs (for Affymetrix 500*k* arrays) genotyped on 100 individuals. Note that this step is designed to be applied chromosome by chromosome since it uses the LD measure as a similarity.

The second step of the proposed algorithm consists in model selection via the Gap statistic. Using the Gap statistic for estimating the number of groups requires the constrained hierarchical clustering algorithm to be applied to *B* reference data sets of the same size as **X**. Thus the Gap-step is *B* times longer than the constrained clustering algorithm and is the computational bottleneck of the method since the complexity of the clustering is quadratic in the number of markers. However the parallelization of the model selection procedure is straightforward.

### Competing methods

Various approaches have been proposed to select causal SNPs from GWAS data. The method described in Section ‘A three-step method for GWAS’ is compared to two groups of methods:

- three methods that do not explicitly take a block-structure information into account: SMA, and two penalized regression approaches: Lasso [6] and Elastic-Net [7].
- two methods that do explicitly take the block-structure information into account: the haplotype association module of the PLINK genome association analysis tool [23], and the Group Lasso applied to the true SNP groups. The latter approach cannot be applied in practice, but is very useful to analyze the contribution of the different steps of the proposed method. We will refer to this approach as the “oracle Group Lasso”.

**Single Marker Analysis** In the standard SMA, for each variable *X*_.*j*_, we fit a single-predictor equation **y**=*β*_0_+*β*_*j*_ **X**_·*j*_ and a *p*-value from a *t*-test against an intercept-only model is calculated.

**Multi-variable approaches** The Lasso [6] is an efficient sparse variable selection model in high-dimensional problems. The estimator of Lasso, denoted by $\hat {\boldsymbol {\beta }}_{\text {lasso}}$ is defined as:

$$ \begin{array}{rcl}{\widehat{\boldsymbol{\beta}}}_{\mathrm{Lasso}}& =& \arg\ { \min}_{\beta \in {\mathbb{R}}^p}\left|\right|\mathbf{y}-\mathbf{X}\boldsymbol{\beta } \left|\right|{}_2^2+\lambda \left|\right|\boldsymbol{\beta} \left|\right|{}_1,\end{array} $$

where ||.||_1_ denotes the *ℓ*_1_ norm and *λ* is a regularization parameter. Thanks to the *ℓ*_1_ penalty, the Lasso encourages sparsity by setting many regression coefficients for irrelevant SNPs to exactly zero. However, this method does not incorporate any information on correlation structure between predictors, and tends to select only one variable in each group of correlated variables. In order to overcome this limitation, other methods have been proposed, including the Elastic-Net [7]. The estimator of Elastic-Net is denoted by $\hat {\boldsymbol {\beta }}_{\text {EN}}$ and defined as:

$$ \begin{array}{rcl}{\widehat{\boldsymbol{\beta}}}_{\mathrm{EN}}& =& \arg\ { \min}_{\beta \in {\mathbb{R}}^p}\left|\right|\mathbf{y}-\mathbf{X}\boldsymbol{\beta } \left|\right|{}_2^2+{\lambda}_1\left|\right|\boldsymbol{\beta} \left|\right|{}_1+{\lambda}_2\left|\right|\boldsymbol{\beta} \left|\right|{}_2^2\end{array} $$

where *λ*_1_ and *λ*_2_ are two regularization parameters. Like the Lasso, the Elastic-Net simultaneously performs automatic variable selection and continuous shrinkage. Unlike the Lasso, the Elastic-Net includes a ridge (*ℓ*_2_) penalty which tends to select groups of correlated variables. Therefore, the Elastic-Net incorporates some prior information regarding the block structure of the data. However, unlike the proposed method, it does not take advantage of the fact that in the particular case of GWAS, LD blocks are adjacent along the genome. In this paper, we chose a large value for the ridge parameter (*λ*_2_=0.8) in order for the Elastic-Net estimate to be substantially different from the Lasso estimate (which corresponds to *λ*_2_=0).

**Haplotype association** This competing grouping strategy includes 4 steps, the first 3 being performed using the PLINK genome association analysis tool. The first step consists in inferring the LD blocks following the confidence intervals procedure [24]. Then within each LD block, haplotypes are estimated using an accelerated EM algorithm similar to the partition/ligation method [25]. In the third step, haplotype-specific tests (with 1 degree of freedom) for a quantitative trait are performed with PLINK using the option *–hap-assoc*. Finally, we define a block-adjusted *p*-value by performing a (Bonferroni) Family-Wise Error Rate correction within each block. The *p*-value of a SNP is then defined as the adjusted *p*-value of the block it belongs to.

### Performance evaluation

Our performance assessment aims at evaluating the ability of our proposed method to retrieve causal SNPs. Performance is evaluated using partial Areas Under the Curve (AUC) of the Receiver Operator Characteristics (ROC) curve. This measure will be denoted by pAUC. We first evaluate, for each method, the True Positive Rate (TPR) and False Positive Rate (FPR) for a grid of underlying regularization parameter values and for each simulation in order to obtain a ROC curve. Then we calculate the pAUC in the range FPR ∈[0,*l* *i* *m*] for each ROC curve, where *lim* is defined as the maximum value of FPR below which the ROC coordinates of all methods are well defined.

### SNP and block-level evaluation

**Figure 2:**
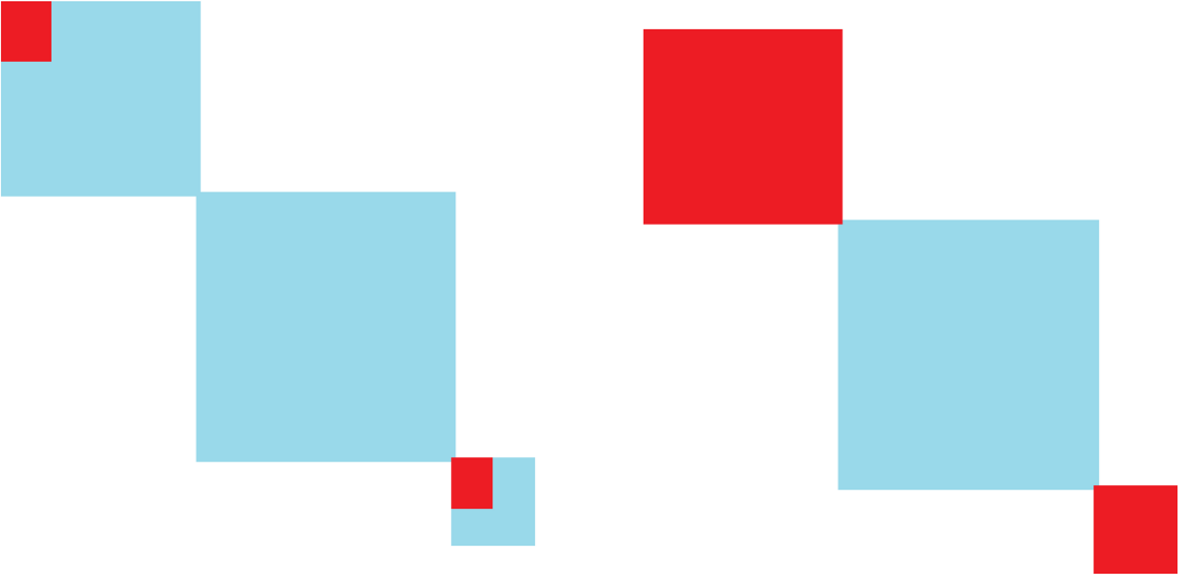
Schematics of covariance matrices for illustration of the proposed definition of “causal SNPs” (red area in the left panel) and “block-associated SNPs” (red area in the right panel) on a toy example with *p*=12 SNPs in 3 blocks of size 4, 6, and 2, respectively.

A SNP may be detected by a given method either because it is a causal SNP, that is truly associated with the phenotype, or because it is in LD with such a causal SNP. This issue is intrinsic to the design of GWAS and thus requires adapted definitions of true and false positives. A relevant recent contribution is the recently-introduced notion of “threshold-specific FDR” (tFDR) [4]. tFDR relies on an alternate definition of true positives that incorporates not only “causal true positives” but also “linked true positives”. In a similar spirit, we consider two definitions of associated SNPs in our simulation setting. We define a *causal SNP* as a SNP that is simulated with a non-zero regression parameter, and a *block-associated SNP* as a predictor that is not a causal marker but simulated in the same LD block that a causal SNP. This is illustrated by Figure 2. Importantly, and contrary to tFDR, our definition of a block-associated SNP does not depend on a correlation threshold.

Therefore, we consider two types of evaluation differing in their objective. In the SNP-level evaluation (left panel in Figure 2), the statistical unit considered is the SNP, and a true positive (in red) is the discovery of a causal SNP; the discovery of any other SNP (in blue) is considered as a false positive. In the block-level evaluation (right panel in Figure 2), the statistical unit considered is the LD block, and a true positive (in red) is the discovery of a block-associated SNP; the discovery of any other SNP (in blue) is considered as a false positive. Given these definitions, we expect better results from the three classical approaches (SMA, Lasso, and Elastic-Net) for the SNP-level evaluation, and better results from the group-based methods for the block-level evaluation.

### Simulation settings

Our simulation setting is adapted from [26]. For all *i*∈{1,…,*n*},**X**_*i*·_ is generated from a p-dimensional multivariate normal distribution whose covariance matrix is block-diagonal. If *j*≠*j* ^′^ are in the same group, $\phantom {\dot {i}\!}cov(\mathbf {X}_{\cdot j}, \mathbf {X}_{\cdot j'})=\rho $ else $\phantom {\dot {i}\!}cov(\mathbf {X}_{\cdot j}, \mathbf {X}_{\cdot j'})=0$. Then, we set *X*_*ij*_ to 0,1 or 2 according to whether *X*_*ij*_<−*c*, −*c*≤*X*_*ij*_≤*c* or *X*_*ij*_>*c*, where *c* is a threshold determined for producing a given minor allele frequency. For example, choosing *c* as the first quartile of a standard normal distribution corresponds to fixing the minor allele frequency of the corresponding SNP to 0.5. The associated continuous phenotype vector is finally generated according to the linear regression model:

$$ \mathbf{y}=\mathbf{X}\boldsymbol{\beta} + \boldsymbol{\epsilon}, $$

where $\boldsymbol {\epsilon } \in \mathbb {R}^{n}$ is a gaussian error term.

## Results and discussion

### Results on simulated data

**Figure 3:**
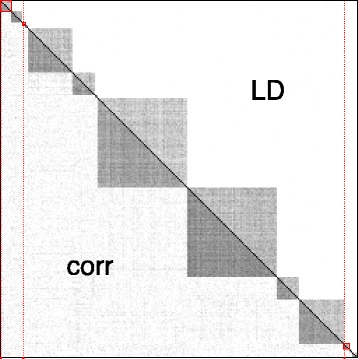
Blockwise dependency for a simulation run, with *ρ* = 0.4, using the same representation and color scale as in Figure 1. The average *r* ^2^ within LD block is approximately 0.2. Red dots correspond to causal SNPs. The blocks in which they are located are highlighted by red squares.

We set *n*=100 and *p*=2,048, with 192 groups of sizes 2,2,4,8,16, and 32, replicated 32 times. The ordering of the groups is drawn at random for each simulation. Figure 3 illustrates the type of dependency structure that is obtained in this setting, using the same type of representation as in Figure 1.

In our simulation, the difficulty of the problem is calibrated according to the coefficient of determination *R* ^2^ of the model, that is, the ratio of the variance explained by the model to the total variance. This coefficient quantifies the ability of a multi-variable model to explain the phenotype using the combined effect of all the relevant markers. It is also called the total heritability *h* ^2^ in the context of genetics [4]. This coefficient is not to be mistaken with the squared Pearson linear correlation coefficient *r* ^2^ between the phenotype and the genotypes of a single marker. Thus, in our simulation setting, the absolute value of the regression coefficients of causal SNPs does not influence the performance of the methods. In the experiments reported below, the regression coefficients of the causal SNPs were randomly set to 1 or −1, and to 0 for all other SNPs; *R* ^2^ is set to 0.2, which appeared to be a realistic value for GWA studies in comparison with the number of individuals *n*=100. The other parameters of the simulation are the within-LD-block correlation coefficient *ρ*, the number causalSNP of causal SNPs and the size sigBlock of the associated block.

We have performed an extensive simulation study, where causalSNP ∈{1,2,4,6,8} and sigBlock ∈{2,4,8,16,32}. We report average pAUC across 300 simulation runs. We mainly focus on cases where the correlation coefficient *ρ*∈{0.2,0.4} as these values yield an average LD within a block that is consistent with what is typically observed in real data (see Figure 1).

#### Block-level versus SNP-level evaluation

**Figure 4:**
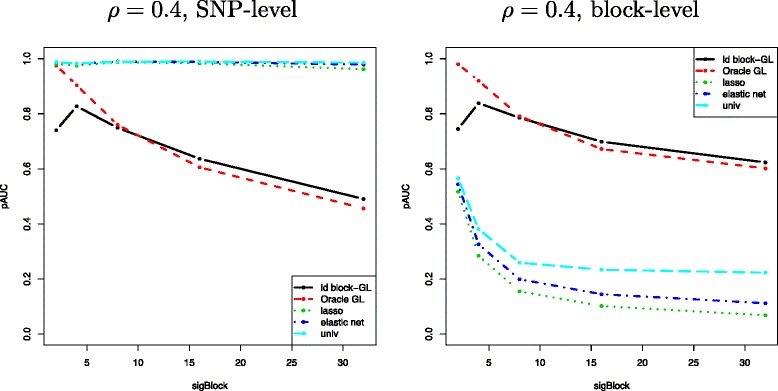
The mean pAUC versus the size of the LD block containing a single causal SNP sigBlock for the proposed method (“ld block-GL”, black solid lines), oracle Group Lasso (dashed red lines), Lasso (dotted green lines), Elastic-Net (dash-dotted blue lines) and SMA (“univ”, dashed light blue lines), for *ρ* = 0.4. Left: SNP-level evaluation. Right: block-level evaluation.

We consider a setting where a single SNP is truly associated with the phenotype. Figure 4 displays the pAUC versus the size sigBlock of the “associated block” (that is, the LD block containing the causal SNP) for both SNP- and block-level evaluations. With SNP-level evaluation (left panel), group-based approaches are outperformed by the three competitors, and increasingly so as the size of the associated block increases. This is mainly due to the high number of false positive SNPs generated by the group selection. Indeed, selecting a group with only one causal SNP causes all other SNPs of the group to be declared as false positives. Conversely, with group-level evaluation (right panel of Figure 4), group-based methods show a clear superiority, showing that multi-variable SNP-based methods (Lasso or Elastic-Net) are generally unable to select all of the causal SNPs due to the presence of correlation between the SNPs of the block. The poor performance of Lasso under correlated designs is not new [7], but Figure 4 suggests that the proposed approach even outperforms Elastic-Net. Although the Elastic-Net has been designed specifically for correlated designs and has recently been shown to have good performance in GWAS [4], it seems that it does not take full advantage of the characteristic block structure of the predictors in GWAS.

As the size of the associated block increases, the performance of all methods decrease. Indeed, for a given level of within-block correlation (here, *ρ*=0.4), the larger the size of the block, the more diluted the information about the causal SNP becomes. Thus, a larger LD block in our simulation setting results in a more difficult problem. This increase in complexity explains the general decrease in performance. This decrease in performance is more severe for the Group lasso. Indeed, it tends to select small groups of SNPs because its default penalty increases with block size. The drop in performance of the proposed approach compared to that of the “oracle” Group Lasso for sigBlock ∈{2,4} is discussed in the next subsection when assessing the efficiency of the block inference step.

In the remainder of this section, we focus on SNP-level evaluation, which is *a priori* more favorable to SNP selection methods than to group selection methods. We are interested in comparing the methods under this evaluation setting which is particularly challenging for the proposed approach.

#### Efficiency of LD block inference

**Figure 5:**
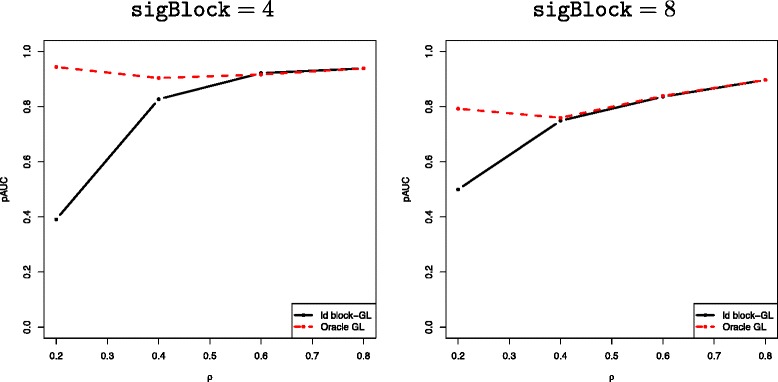
Average pAUC versus correlation level *ρ* for the proposed method (“ld block-GL”, black solid lines) and an oracle version where the LD blocks are assumed to be known (dashed red lines), for sigBlock ∈{4,8}.

The goal of this section is to quantify the inference of the LD blocks (the first two steps in Section ‘A three-step method for GWAS’) on the global performance of the proposed method. In order to do so, we compare the performance of the proposed method to that of the “oracle” version where the Group Lasso is applied to the true LD blocks, that is, those defined by the simulation settings. Figure 5 displays the mean pAUC versus the correlation level for both methods. When the level of correlation is less than 0.4, we note that the proposed approach is outperformed by the “oracle” Group Lasso. In fact, for low correlation levels, the block inference procedure tends to under-estimate the number of blocks leading to an estimated group structure with big blocks and thus a high number of false positives selected by the Group Lasso. However, the difference between the performance of the two group-based methods becomes insignificant when the level of correlation is above 0.4 and when the size of the associated block is greater than 4. This indicates that the proposed LD block inference method, which combines constrained clustering and model selection, efficiently captures the underlying dependency structure in this case.

#### Influence of the number of causal SNPs per block

**Figure 6:**
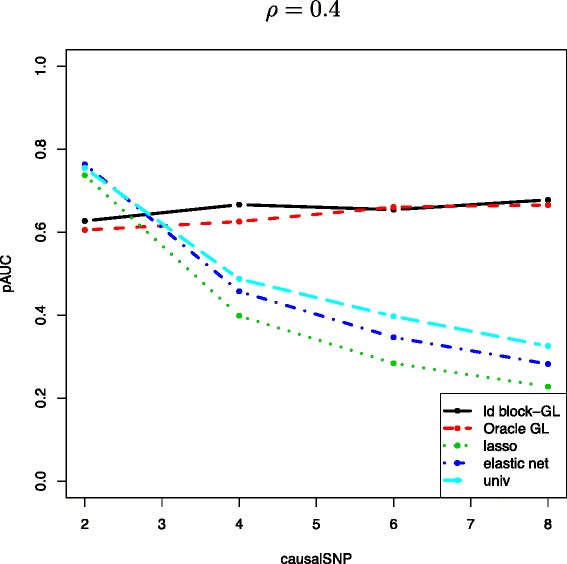
The mean pAUC as a function of the number of causal SNPs causalSNP within a block of size 8, for the proposed method (“ld block-GL”, black solid lines), oracle Group Lasso (dashed red lines), Lasso (dotted green lines), Elastic-Net (dash-dotted blue lines) and SMA (“univ”, dashed light blue lines), for *ρ* = 0.4.

We investigate the robustness of the 5 approaches to the parameter causalSNP, that is, the number of relevant variables within a block of size 8. Figure 6 displays the pAUC as a function of causalSNP for *ρ*=0.4.

These results illustrate the robustness of the proposed group approach to an increasing number of causal SNPs, which is not the case of its 3 competitors. Indeed, the performance of the group strategies remain constant when that of the classical approaches deteriorate significantly as soon as the number of relevant SNP within the block exceeds 2. More specifically, the Group Lasso selects the associated block of 8 SNPs for both correlation levels. On the contrary, the Lasso fails to recover the true relevant SNPs if there are correlations among the variables. As expected, the Elastic-Net performs a little better than the Lasso when the correlation structure is strong enough for the grouping effect of this model to be effective (*ρ*≥0.4).

#### Influence of the Minor Allele Frequency distribution

Our simulation model adapted from [26] allows to reproduce the group-structured correlation that characterizes the GWAS data (see Figure 3). However, as noted by a reviewer, fixing the cutoff parameter *c* at the first quantile of the standard normal distribution as in [26] generates unrealistic Minor Allele Frequency (MAF) distributions. To address this point, we simulated genotype matrices where the MAF of the SNPs are uniformly sampled between 0.05 and 0.5. This roughly corresponds to the MAF distribution observed in a real GWA study [11], and MAF =0.05 is a commonly-used threshold to partition variants into rare and common.

We then performed the same simulation study presented above adapting the dimension parameters to the new range of MAF. Specifically, we used *n*=1, 000 in order for variants with a low MAF to be observed frequently enough. Accordingly, the *R* ^2^ ratio was lowered to 0.01 in order for the difficulty of the problem to be similar. The number of markers was increased to *p*=4, 096 in order to maintain *p*≫*n*. Finally, groups of sizes 2,2,4,8,16, and 32 were replicated 64 times, yielding a total of 384 groups.

The results and conclusions are almost identical to those of the previous subsections (see Additional files 1 and 2). Firstly, for the scenario with an isolated causal SNP as in Section ‘Block-level versus SNP-level evaluation’ and for the scenario with an increasing number of causal markers as in Section ‘Influence of the number of causal SNPs per block’, the ordering of the performance of all the methods remained unchanged with a general increase for all the approaches due to the less stringent high-dimensionality ratio *n*/*p* compared to the ratio used in the previous subsections. Secondly, the first two steps of the proposed approach were able to perfectly retrieve the underlying block structure, even with low values of the correlation. In contrast, performance curves in scenario ‘Efficiency of LD block inference’ were superimposed only for *ρ*≥0.4. This difference can be explained by the fact that increasing the number of individuals *n* led to a more salient LD block structure.

### Results on semi-simulated data

In order to control the causal SNPs while considering a realistic dependance structure among the SNPs, we used semi-simulated data, where the genotypes come from a real GWA study and the phenotypes are simulated using the linear model presented in Section ‘Simulation settings’ with pre-determined causal SNPs. This type of simulation allows to study a data set with a real linkage disequilibrium structure while having a ground truth. The genotype data correspond to the first *p*=2, 048 SNPs of chromosome 22 for *n*=100 individuals from a GWA study on HIV [11]. This data set is described in more detail in Section ‘Analysis of HIV data’. The LD block structure was firstly inferred using the first steps of the two group-based approaches:

- CHC-Gap : the proposed constrained hierarchical clustering followed by the Gap statistic.
- CI : the default confidence intervals method used in PLINK.

**Figure 7:**
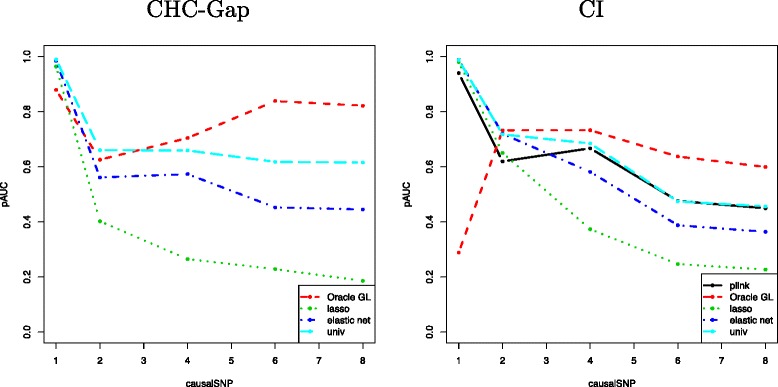
The mean pAUC as a function of the number of causal SNPs causalSNP within a block of size 8, for the haplotype association method (“plink”, black solid line), oracle Group Lasso (dashed red lines), Lasso (dotted green lines), Elastic-Net (dash-dotted blue lines) and SMA (“univ”, dashed light blue lines). Left: The LD blocks inferred using CHC-Gap. Right: The LD blocks inferred using CI.

The procedure CHC-Gap estimated 225 blocks and the procedure CI inferred 993 blocks including 555 blocks of size 1 (single SNPs). Similar to the previous simulation study, 300 continuous phenotypes were generated by increasing the number of relevant variables causalSNP within a block of size 8. Figure 7 displays the pAUC as a function of causalSNP.

Given the blocks estimated with CHC-Gap, we compared the performance of the proposed method to that of the non-grouping approaches (left panel of Figure 7). As in Section ‘Block-level versus SNP-level evaluation’, for causalSNP ∈{1,2}, the proposed approach is outperformed by its competitors because of the high number of false positives generated by the group selection. Conversely, the performance of the competing methods deteriorate significantly as soon as causalSNP >2 which is not the case of the Group Lasso. This result is also consistent with those obtained in Section ‘Influence of the number of causal SNPs per block’.

Similarly, given the block structure inferred with CI, we investigated the robustness of the oracle Group Lasso, the haplotype association approach and the 3 non-grouping methods to the parameter causalSNP (right panel of Figure 7). Comparing haplotype association and Group Lasso approaches, we observe a difference of performance when one unique causal SNP is included in a block. The drop in performance of the Group Lasso is due to the difference in the block structure: as explained in Section ‘Block-level versus SNP-level evaluation’, the Group Lasso penalty increases with block size, making it difficult for this method to select the correct block in presence of many smaller blocks. In practice, this is not problematic as the block selection step in the proposed approach yields larger blocks. On the contrary, the haplotype association method performs a *p*-value correction that takes the block structure into account, but the *p*-value of the causal SNP is so small that the adjustment hardly reduces the significance of the block. Furthermore, as in Section ‘Simulation settings’, it is remarkable that Group Lasso outperforms competing approaches as soon as causalSNP >2 even for SNP-level evaluation.

The consistency between the results of Sections ‘Results on simulated data’ and ‘Results on semi-simulated data’ suggests that the simulation setting used in Section ‘Results on simulated data’ efficiently mimics a realistic genotyping data set.

### Analysis of HIV data

#### Data set

The HIV data set consists of *p*=20,811 SNPs genotyped for *n*=605 Caucasian subjects and the plasma HIV-RNA level as phenotype. It corresponds to the phenotype and the genotype data related to the chromosome 6 of the GWA study conducted by [11]. A small number of SNPs were discarded from the study because they generated undefined values of LD. The filtered data set thus contained 20,756 SNPs. Missing values were imputed using the Bioconductor R package snpStats [21]. For the proposed approach, this imputation was performed after the constrained clustering described in Sections ‘Inference of LD blocks from genotypes’ and ‘Estimation of the number of groups’, as the proposed constrained clustering algorithm handles missing values. The same data set was used to perform the haplotype association approach. Each of the compared models was adjusted for the gender of the patient.

#### Block inference

**Figure 8:**
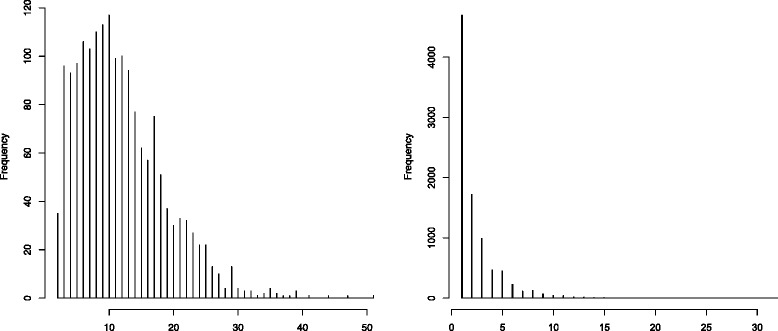
Histograms of the estimated block sizes of the HIV data. Left: the histogram of the block sizes estimated by the first 2 steps of the proposed method. Right: the histogram of the block sizes estimated by the first step of the haplotype association approach.

The first step of inferring the LD blocks applied to the HIV data estimated 1,756 blocks with *B*=500 null reference data sets generated in the Gap statistic algorithm. The distribution of the sizes of the obtained blocks is represented in the histogram of Figure 8 (left panel). The median block size is close to 10, and the size of the vast majority of blocks is less than 30. The first step of the haplotype association method estimated 9,003 haplotype blocks including 4,699 single SNPs. The size distribution of the obtained blocks is represented in the histogram on the right panel of Figure 8. Unlike the LD structure inferred by the proposed approach, the haplotype blocks are much smaller with an average block size of 2.

#### Results on HIV data

**Figure 9:**
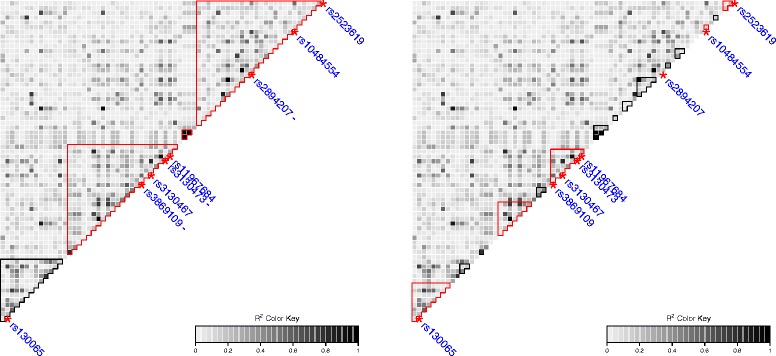
A linkage disequilibrium (*r* ^2^) plot with the inferred block structures (black and red contour lines) for a set of 68 contiguous SNPs located on the MHC region. Left: within the structure inferred by the proposed method, the blocks selected by the Group Lasso are delimited with a red contour line. The SNPs selected by SMA are plotted with a red star (*), and the SNPs missed by Lasso with a blue dash (**-**). Right: within the structure inferred by the haplotype association method, the blocks selected by the competing method are delimited with a red contour line.

We were able to reproduce the results of [11]: the SNPs identified by SMA correspond to the 15 SNPs selected by [11] at a target False Discovery Rate (FDR) level of 25*%*. Most of these SNPs are located in the major histocompatibility complex (MHC) region 6p21. A linkage disequilibrium plot of a set of 68 contiguous SNPs within this region is represented in Figure 9. The SNPs marked with a red star (*) are those selected by SMA. The first 20 SNPs selected by the Lasso are the same as those selected by the univariate model except for 3 SNPs; the names of these 3 SNPs are marked with blue dashes (**-**) in the left panel of Figure 9.

The local block structures inferred by both the clustering and model selection steps of the proposed method and the competing haplotype association method are also highlighted in this figure (contour lines). The mean LD within the largest two blocks of the left panel is *r* ^2^=0.41 and *r* ^2^=0.55, respectively. The Lasso was able to recover two of the four SNPs identified by [11] in the first block, and two of the three SNPs identified by [11] in the second block. This is consistent with the fact that the Lasso is not designed to select correlated variables, as already discussed in Section ‘Competing methods’.

Among the four blocks inferred by the proposed method in this region, the three blocks with a red contour line are among the first 15 blocks selected by the Group Lasso (see Additional file 3 for a Manhattan plot). Almost all of them are of size more than 10 SNPs, except for the two blocks containing 3 and 4 SNPs already identified by [11], as displayed in Figure 9. Each of the 8 remaining SNPs selected by SMA are located in a different LD block of average size around 18 SNPs. The fact that these SNPs have not been detected by the Group Lasso is consistent with the results of our simulation data. Indeed, Figure 4 showed that the Group Lasso tends to select small groups of SNPs because of its default penalty.

**Figure 10:**
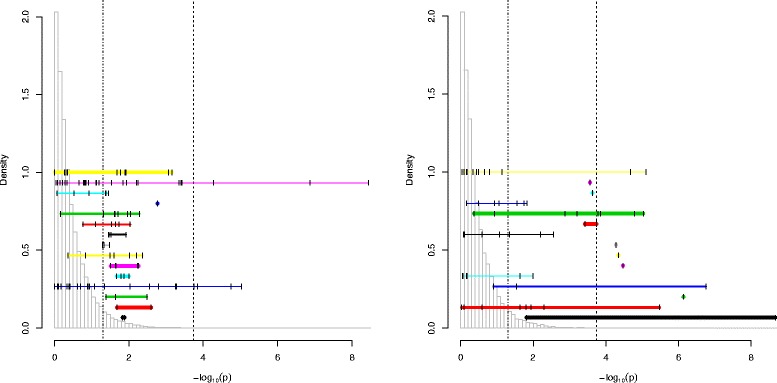
A comparison between the results of [11] and the grouping methods on HIV data. The gray histogram represents the distribution of the (− log10-transformed) SMA *p*-values obtained by [11]. Each of the first 15 blocks selected by the proposed approach (left panel) and the first 15 blocks selected by the haplotype association method (right panel) are represented by a colored horizontal segment ranging from the smallest to the largest SMA *p*-value of the block. Vertical black segments indicate SMA *p*-values of each SNP in these LD blocks. Vertical lines highlight the significance threshold used in [11] (dashed line) and the standard (non multiplicity-corrected) level of 0.05 (dash-dotted line).

Contrary to the Lasso or the Elastic-Net, the proposed approach detected groups of SNPs that had not been identified by [11]. Some of these groups of SNPs may contain interesting candidates, as further discussed below in the description of Figure 10.

Similarly to the proposed method, we focused on the first 15 blocks (including single SNPs) selected by the haplotype association approach. The 5 blocks selected by the haplotype association method in the same region represented in Figure 9 are represented with a red contour line. The competing approach was able to recover all of the 7 SNPs identified by [11] and located in this region. However, it detected one group of SNPs that had not been identified in the previous study. This difference could be due to strong LD (*r* ^2^=0.81) between the SNPs of this block and the SNPs of the block containing 4 markers previously identified as associated with the phenotype.

Each of the first 15 LD blocks selected by the two grouping strategies are represented as a colored horizontal segment in Figure 10, where the *x* axis corresponds to the (− log10-transformed) SMA *p*-values obtained by [11].

For the haplotype association approach (right panel of Figure 10), 6 of the 15 blocks consist of a single SNP, that had already been identified in [11]. Moreover, for several of the 15 LD blocks selected by the proposed approach (left panel of Figure 10), *all of the SMA p-values of the block* are smaller than the (non multiplicity-corrected) 0.05 level (vertical dash-dotted line at − log10(*p*)=1.3). Therefore, although we do not claim that all of these groups of SNPs are relevant to HIV, we believe that some of them might contain interesting candidates. The dashed vertical line highlights the significance threshold used in [11]. Therefore, the 4^th^ and 14^th^ blocks which cross the vertical dotted line correspond to the two largest blocks in the left panel of Figure 10, which respectively contain 4 and 3 SNPs previously identified by [11]. We also believe that Figure 10 is an interesting diagnostic plot to pinpoint candidate groups of SNPs associated with the disease. Further replication or meta-analysis work would be required to confirm the association between these novel candidates and the phenotype.

## Conclusions

In this paper, we have proposed a three-step approach that takes into account the biological information of the linkage disequilibrium between variables by firstly inferring LD blocks, then estimating the number of such blocks, and finally performing Group Lasso regression on these inferred groups. This method is implemented as an R package. Although we have used a continuous phenotype in our simulations and applications, the approach described in this paper can be extended to the study of categorical phenotypes, by using the logistic version of each regression model.

We have demonstrated using simulations that the proposed approach efficiently retrieves the underlying block structure for realistic levels of LD between SNPs. Moreover, state-of-the-art SMA and penalized regression approaches Lasso and Elastic-Net are outperformed by our proposed method for the purpose of identifying *blocks containing causal SNPs*. We have argued that selecting *blocks* (rather than individual SNPs) associated with a phenotype is a sensible goal in the GWAS context, where the proportion of heritability explained by individual SNPs is generally low. Interestingly, although the proposed method can only select groups of SNPs and not individual SNPs, our results on simulated data suggest that this approach performs better than state-of-the-art approaches in terms of selection of causal SNPs as soon as the number of associated SNPs within the same LD block exceeds 2.

We have also applied this method to semi-simulated data with a real genotype matrix and a simulated phenotype. As soon as the number of causal markers within a block exceeds 2, the proposed approach shows remarkable performance compared to non-grouping classical strategies, and to an haplotype association method that explicitly takes the block structure information into account. This result suggests that the proposed method is adapted to a real linkage disequilibrium structure.

Finally, an application of this method to HIV data illustrates the ability of the method to (i) partly recover the signal identified by single-locus methods, and (ii) pinpoint previously overlooked associations. We believe that these results demonstrate the relevance of the approach, and thereby illustrate the importance of tailored integration of biological knowledge in high-dimensional genomic studies such as GWAS.

A limitation of our proposed method is that it does not provide a significance assessment for the selected groups. Deriving reliable *p*-values for regression coefficients in high-dimensional, correlated settings is a challenging research area in the machine learning and statistics fields in general [27,28].

However, even if such *p*-values could be obtained for the groups inferred by our proposed method, we would like to emphasize that providing an interpretable multiple testing risk assessment in GWAS would remain a difficult question. Although several multi-SNP tests have been proposed to assess the significance of SNP groups [5,29], no fully satisfactory strategy allows the control of standard multiple testing error rates such as the Family-Wise Error Rate (FWER) or the False Discovery Rate (FDR). Indeed, the presence of correlation among explanatory variables makes causal SNPs indistinguishable from their “neighbors”. This issue is not specific to a particular inference method, but intrinsic to the design of GWAS. Therefore, we believe that it should be addressed by adapting the definitions of true and false positives. In the present paper, we have considered two types of risk evaluation at different *genomic scales*: SNP-level and block-level evaluations. An alternative strategy in a similar spirit was recently proposed [4]. Both strategies rely on a prior definition of the scale of the signal of interest. For future work, we would like to develop an evaluation strategy and an associated inference method that adapts to this scale. A possible direction is to adapt the notion of hierarchical testing of variable importance [30,31] to the specific context of GWAS.

## Additional files

Additional file 1 Figure S1. Simulation results with realistic MAF for the scenario with an isolated causal SNP.

Additional file 2 Figure S2. Simulation results with a MAF uniformly distributed in [0.05,0.5] for the scenario with an increasing number of causal SNPs within a block of size 8.

Additional file 3 Figure S3. Manhattan plot of the HIV data results.

## Abbreviations

AUC: Area under the curve
BALD: Blockwise approach using linkage disequilibrium
FDR: False discovery rate
FWER: Family-wise error rate
GWAS: Genome-wide association studies
HIV: Human immunodeficiency virus
LD: linkage disequilibrium
MAF: Minor allele frequency
SMA: Single marker analysis
SNP: Single nucleotide polymorphism
