# Supplementary material for: Performance of a blockwise approach in variable selection using linkage disequilibrium information
Source: BMC Bioinformatics. 2015 May 8;16:148. doi: 10.1186/s12859-015-0556-6 (PMC4430909; doi:10.1186/s12859-015-0556-6)
Supplement: Additional file 1 — Figure S1. Simulation results with realistic MAF for the scenario with an isolated causal SNP. [file 12859_2015_556_MOESM1_ESM.pdf]

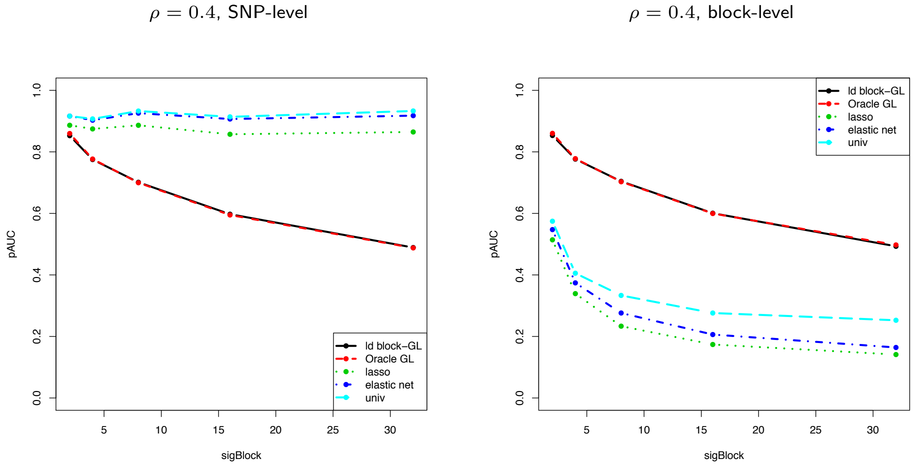

Figure S1 The mean pAUC versus the size of the LD block containing a single causal SNP `sigBlock` for the proposed method ("ld block-GL", black solid lines), oracle Group Lasso (dashed red lines), Lasso (dotted green lines), Elastic-Net (dash-dotted blue lines) and SMA ("univ", dashed light blue lines), for $\rho = 0.4$. Left: SNP-level evaluation. Right: block-level evaluation.
