# Supplementary material for: Performance of a blockwise approach in variable selection using linkage disequilibrium information
Source: BMC Bioinformatics. 2015 May 8;16:148. doi: 10.1186/s12859-015-0556-6 (PMC4430909; doi:10.1186/s12859-015-0556-6)
Supplement: Additional file 2 — Figure S2. Simulation results with a MAF uniformly distributed in [0.05,0.5] for the scenario with an increasing number of causal SNPs within a block of size 8. [file 12859_2015_556_MOESM2_ESM.pdf]

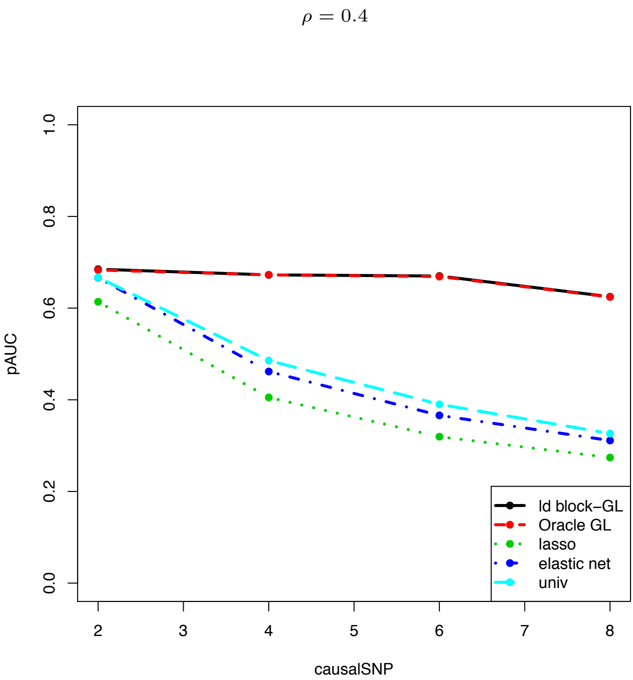

Figure S2 The mean pAUC as a function of the number of causal SNPs `causalSNP` within a block of size 8, for the proposed method ("ld block-GL", black solid lines), oracle Group Lasso (dashed red lines), Lasso (dotted green lines), Elastic-Net (dash-dotted blue lines) and SMA ("univ", dashed light blue lines), for $\rho = 0.4$.
