# Supplementary material for: Performance of a blockwise approach in variable selection using linkage disequilibrium information
Source: BMC Bioinformatics. 2015 May 8;16:148. doi: 10.1186/s12859-015-0556-6 (PMC4430909; doi:10.1186/s12859-015-0556-6)
Supplement: Additional file 3 — Figure S3. Manhattan plot of the HIV data results. [file 12859_2015_556_MOESM3_ESM.pdf]

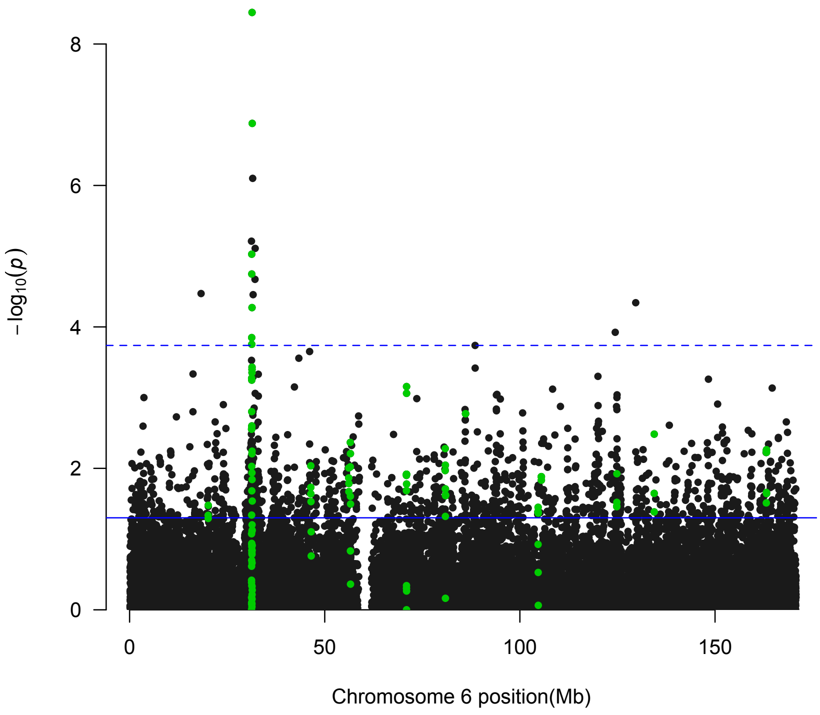

Figure S3 Manhattan plot for the HIV data results. The markers highlighted in green correspond to the SNPs of the first 15 blocks selected by the proposed approach on the MHC region.
